# Typhus Fever

**Published:** 1850-03

**Authors:** 


### SELECTIONS

FROM

### AMERICAN AND FOREIGN JOURNALS.

---

*Typhus Fever.*—In cases which last for a lengthened period, in which the pulse indicates the want of stimulants, and where delirious restlessness was present, Dr. Jones Lamprey, of Meath county, Ireland, recommends the use of port wine. The antimonial treatment, recommended by Dr. Graves, was found useful in composing the nervous disorder. Dr. Lamprey says: "Where the petechia were of a very dark color, or threatening to run into a putrid condition, I have found the exhibition of yeast, in combination with camphor and nitric ether, of great service in staying the septic process, besides acting as a powerful stimulant. I cannot speak too highly of the stimulating and antiseptic properties of this mixture. I have seen it remove the dark, livid hue of the skin in this form of fever in a few hours. The following is the formula I have used:—

"℞. Yeast, ℥x., Camphor, ʒss., nitric ether, ℥iv. Mix. An ounce to be taken every hour, or every second or third hour."

[A good deal of experience in the use of this remedy, in the last fifteen years, enables us to speak highly of its merits.—*Ed. W. J. M. & S.*]

